# Aspects of Non-Pharmacological Treatment in Peripheral Arterial Disease

**DOI:** 10.5935/abc.20190208

**Published:** 2019-09

**Authors:** Maria Janieire de N. Nunes Alves, Francis Ribeiro de Souza

**Affiliations:** Instituto do Coração (InCor), Hospital das Clinicas da Faculdade de Medicina da Universidade de São Paulo (HC-FMUSP), São Paulo, SP - Brazil

**Keywords:** Peripheral Arterial Disease/physiopathology, Peripheral Arterial Disease/drug therapy, Aged, Intermittent Claudication, Ankle Brachial Index, Exercise, Prevention and Control

Peripheral arterial disease (PAD) is one of the main atherosclerotic diseases in the elderly population^1^ which limits the performance of physical activity. Patients with PAD who have intermittent claudication may have gait impairment, which compromises daily living activities.^2^ In addition, these patients have other comorbidities that may increase cardiovascular risk.^1,3^ Regular exercise is a non-drug treatment recommended for the prevention and treatment of cardiovascular disease. On the other hand, maintaining adherence to a physical training program becomes a major challenge.

The present study^4^ exposes the physical activity pattern of patients with PAD and demonstrates the high rate of a sedentary lifestyle with aging. In fact, the disease itself leads to physical limitation, which in turn is also a worsening factor of the disease, since the recommendation of vigorous physical activity has therapeutic effects that contribute, in addition to the chronic use of arterial vasodilator drugs. In addition, chronic arterial vasodilation due to the use of medications may lead to reduced long-term peripheral flow, as arterial vasodilation promoted by medication further reduces perfusion pressure in the lower limb peripheral muscles and intensifies the low level of physical activity in patients with PAD, according to current physical activity recommendations.

The assessment of the degree of physical activity was performed by a device over a period of 7 days, and the data are relevant in classifying the degree of physical limitation with aging, as well as, demonstrating the significance of sedentary lifestyle, may corroborate to further aggravate the disease and cardiovascular risk. On the other hand, the device does not evaluate or quantify localized muscular resistance training (strength), which may underestimate the results pointed out by the study.

Clearly, the importance of developing strategies for these patients to engage in regular exercise. The main reason for these patients with PAD not progressing with physical training is pain related to increased muscle energy demand, called intermittent claudication, especially in the calf region during walking.^5^ Moderate to vigorous exercise can precipitate the symptoms of intermittent claudication and this becomes a barrier for these patients to engage in physical activity. Thus, low intensity and progressive aerobic exercise can be used as a strategy to delay these symptoms. In addition, patients with intermittent claudication have muscle atrophy, and reduced muscle strength and endurance in the lower limbs increase the fragility of these patients, especially with aging.^6^ In this aspect, strength exercises and peripheral muscular endurance (bodybuilding) should be used.

Although the literature recommends exercises with aerobic characteristics, such as walking, as the main exercise modality for these patients with PAD, muscle overload exercises have also been recommended as part of a physical exercise program in this population.^7,8^

Regular exercise should be used as a non-pharmacological tool for the treatment and prevention of cardiovascular disease in patients with PAD and intermittent claudication, so it is important to develop a specific exercise program, especially to delay the onset of symptoms. intermittent claudication, because, besides preventing the onset of pain and the interruption of exercise, it can be a motivational factor that will imply the adherence of these patients to physical activity.
